# Differences in the gut Firmicutes to Bacteroidetes ratio across age groups in healthy Ukrainian population

**DOI:** 10.1186/s12866-020-01903-7

**Published:** 2020-07-22

**Authors:** Alexander Vaiserman, Mariana Romanenko, Liubov Piven, Vladislav Moseiko, Oleh Lushchak, Nadiia Kryzhanovska, Vitaly Guryanov, Alexander Koliada

**Affiliations:** 1 Institute of Gerontology, Vyshgorodskaya st. 67, Kyiv, 04114 Ukraine; 2 Molecular Genetic Laboratory Diagen, Kyiv, Ukraine; 3 Vasyl Stefanyk Precarpathian National University, Ivano-Frankivsk, Ukraine; 4 PB MEDICOM-IN, Dnipro, Ukraine; 5 Bogomolets National Medical University, Kyiv, Ukraine

**Keywords:** Gut microbiota composition, Firmicutes/Bacteroidetes ratio, Aging, Age-related changes

## Abstract

**Background:**

Gut microbiota plays an important role in physiological and pathological processes of the host organism, including aging. Microbiota composition was shown to vary significantly throughout the life course. Age-related changes in the composition of microbiota were reported in several human studies. In present study, age-related dynamics of phylogenetic profile of gut microbiota was investigated in 1550 healthy participants from Ukrainian population.

**Results:**

Significant changes in the microbiota composition determined by qRT-PCR at the level of major microbial phyla across age groups have been observed. The relative abundance of Actinobacteria and Firmicutes phyla increased, while that of Bacteroidetes decreased from childhood to elderly age. Accordingly, the Firmicutes/Bacteroidetes (F/B) ratio was shown to significantly increase until elder age. In both sexes, odds to have F/B > 1 tended to increase with age, reaching maximum values in elder age groups [OR = 2.7 (95% CI, 1.2–6.0) and OR = 3.7 (95% CI, 1.4–9.6) for female and male 60–69-year age groups, respectively, compared to same-sex reference (0–9-year) age groups].

**Conclusions:**

In conclusion, data from our study indicate that composition of the human intestinal microbiota at the level of major microbial phyla significantly differs across age groups. In both sexes, the F/B ratio tends to increase with age from 0–9-year to 60–69-year age groups. Further studies are needed for a better understanding of mechanisms underlying age-related dynamics of human microbiota composition.

## Background

Accumulating evidence indicates that intestinal microbiota (microbial community inhabiting the gut) is crucially involved in the host organism’s vital functions [1]. The crucial role of the gut microbiota and its metabolites in regulating multiple physiological functions of the host is firmly established [2]. In particular, the intestinal microbiota essentially contributes to human metabolism by providing enzymes which are not encoded by the human genome but play important roles in the breakdown of polysaccharides and polyphenols and also in synthesis of vitamins [3]. Disturbances in gastrointestinal physiology mediated by the loss of microbial diversity or changes in relative abundance of the gut microbial communities are commonly referred to as dysbiosis [4]. Such disturbances caused by disease or aging may impair normal nutrient intake and microbiota functions, while changes in microbiota composition may, in turn, significantly contribute to the age-associated functional decline and various pathological conditions [5]. The disruption of the healthy microbial community may cause systemic pathological conditions such as atherosclerosis, type 2 diabetes and cancer [5, 6].

Both composition of gut microbiota and, accordingly, microbiome (collective genomes of all the microbes inhabiting the gastrointestinal tract) were shown to vary significantly throughout the life course [7–9]. Available evidence suggests that adult-like composition of gut microbiota is established early in life [10–14]. The microbial composition develops into a quite stable adult-like pattern during the first 2–3 years of the child’s life and remains relatively unchanged across the rest of the human life course [15–17]. Unfavorable life events such as diseases, antibiotic treatments and sharp changes in diet may only cause chaotic and transient shifts in diversity, composition and functional features of intestinal microbiome [1]. The most pronounced microbiota changes occur during the transition from adulthood to old age. In elderly (more than 65-year-old) individuals, a reduction in the diversity of microbiome along with greater inter-individual microbiota variations have been observed compared to adult ones [18, 19]. Accumulating evidence from both animal models and human studies indicates that gut microbiota composition plays an important role in the host aging and determines the potential of longevity [8]. In particular, long-term has been shown to promote age-related processes such as systemic inflammation and insulin resistance [20, 21]. The age-related changes in microbiota composition, however, can not necessarily be caused by aging process per se, but they might be also associated with general decline in health status caused by malnutrition or increased need for medications such as non-steroidal anti-inflammatory drugs or antibiotics [18].

The purpose of present study was to investigate whether age-related changes exist in phylogenetic profile of gut microbiota, in particular in the Firmicutes to Bacteroidetes (F/B) ratio, in healthy population of Ukraine. In contrast to previous studies on the topic performed on small-size samples, our study has been performed with a larger sample size (*n* = 1550), allowing to draw more robust conclusions on the age-related dynamics of microbiota composition.

## Results

Significant changes in the gut microbiota composition across age groups have been observed. The relative abundance of Firmicutes phylum tended to increase with age. In the oldest group included in overall analysis (60–69 years), it was 40% higher than in children group (0–9 years) (Table 1). The Bacteroidetes abundance demonstrated the opposite age trend: it was about 80% lower in elderly compared to children. As a result, the F/B ratio tended to increase with age reaching the highest value (1.42) in the 60–69-year age group.

**Table 1:** Median values and 25/75 percentiles of main microbiota phyla across age groups (mixed sexes)

Age group, years	n	Firmicutes		Bacteroidetes		F/B ratio		Actinobacteria		Others	
		Median	IQR	Median	IQR	Median	IQR	Median	IQR	Median	IQR
0–9	153	33.32	22.31–47.12	47.96	35.96–59.18	0.69	0.39–1.32	5.65	4.06–10.29	8.96	5.76–14.06
10–19	93	41.17	22.94–53.16	38.83	25.68–56.21	1.07	0.42–2.06	7.58	4.87–11.28	8.66	6.28–12.7
20–29	256	38.25	26.41–51.04	43.0	28.20–57.55	0.9	0.48–1.8	6.70	4.34–10.66	9.59	5.75–13.83
30–39	451	44.17^a^	29.19–56.21	36.6^a^	21.12–52.0	1.2^a^	0.59–2.62	7.23	4.61–11.77	9.71	5.91–13.22
40–49	321	44.96^a^	28.72–56.63	37.69^a^	21.06–51.85	1.14^a^	0.50–2.81	7.64^a^	5.20–12.43	8.93	4.96–12.6
50–59	190	43.66^a^	29.62–57.9	35.65^a^	19.51–53.63	1.17^a^	0.56–2.73	7.53	4.57–11.69	9.64	5.64–14.55
60–69	67	47.12^a^	35.24–61.41	31.6^a^	20.22–48.53	1.42^a^	0.73–2.78	7.71	4.60–11.54	9.25	4.96–12.52

*IQR* Interquartile range

^a^ Significant difference from the reference group (0–9 yrs) at *p* < 0.01, by Kruskal-Wallis ANOVA followed by post-hoc Dunn’s test for multiple comparisons

The opposite trend to decrease F/B ratio has been observed in a more advanced age group (70+). This age group was not included in the overall analysis due to its small sample size (*n* = 19; among them, 8 men and 11 women). In this age group, the F/B ratio was estimated to be 0.87 (IQR: 0.24–1.53). This ratio was significantly lower than that in the eldest (60–69-year) age group included in the overall analysis and did not differ significantly from that in the children’s (0–9-year) group.

**Fig. 1:**
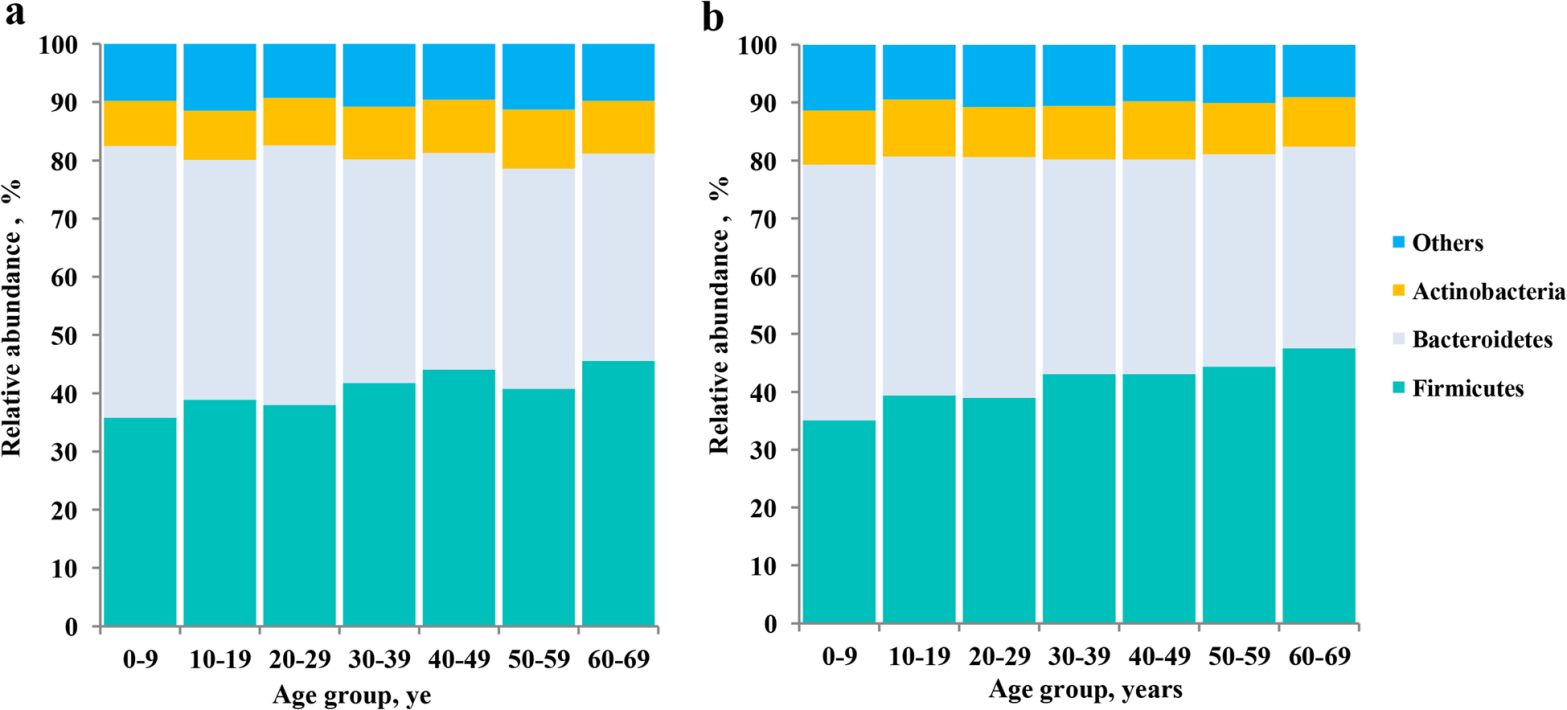
Changes in relative abundance of major gut microbiota phyla across age groups; (**a**) male; (**b**) female

**Fig. 2:**
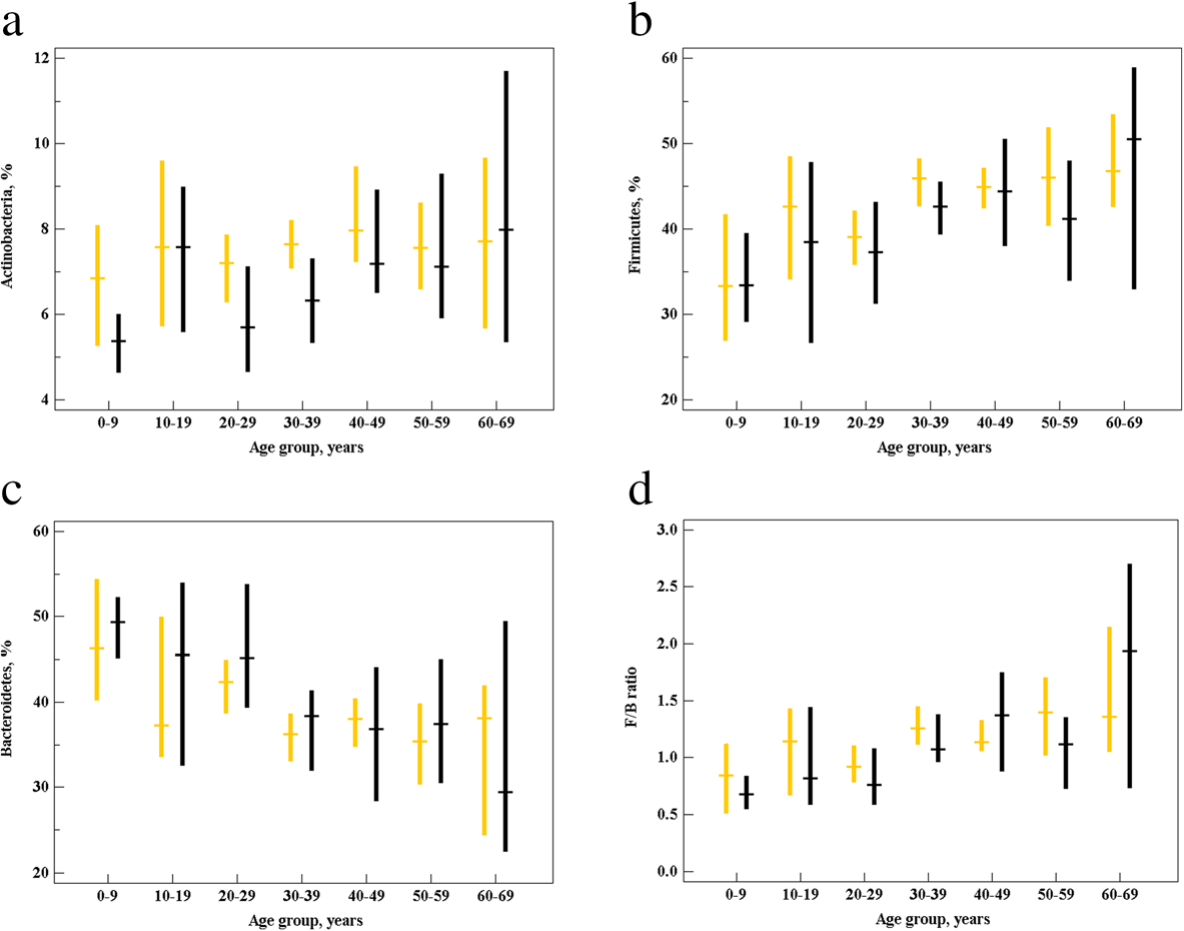
Relative abundance (%) of major gut microbiota phyla: (**a**) Actinobacteria (**b**) Firmicutes, (**c**) Bacteroidetes, and (**d**) F/B ratio. Data are given as median values (horizontal lines) with whiskers indicating 95% confidence intervals (CIs). Female, orange; male, black

The relative abundance of Actinobacteria, similarly to that for Firmicutes, tended to increase with age. This trend, however, was less pronounced than that in Firmicutes. No significant age changes were found for other bacterial groups. Interestingly, the changes observed were quite similar among sexes (see Figs. 1 and 2).

**Fig. 3:**
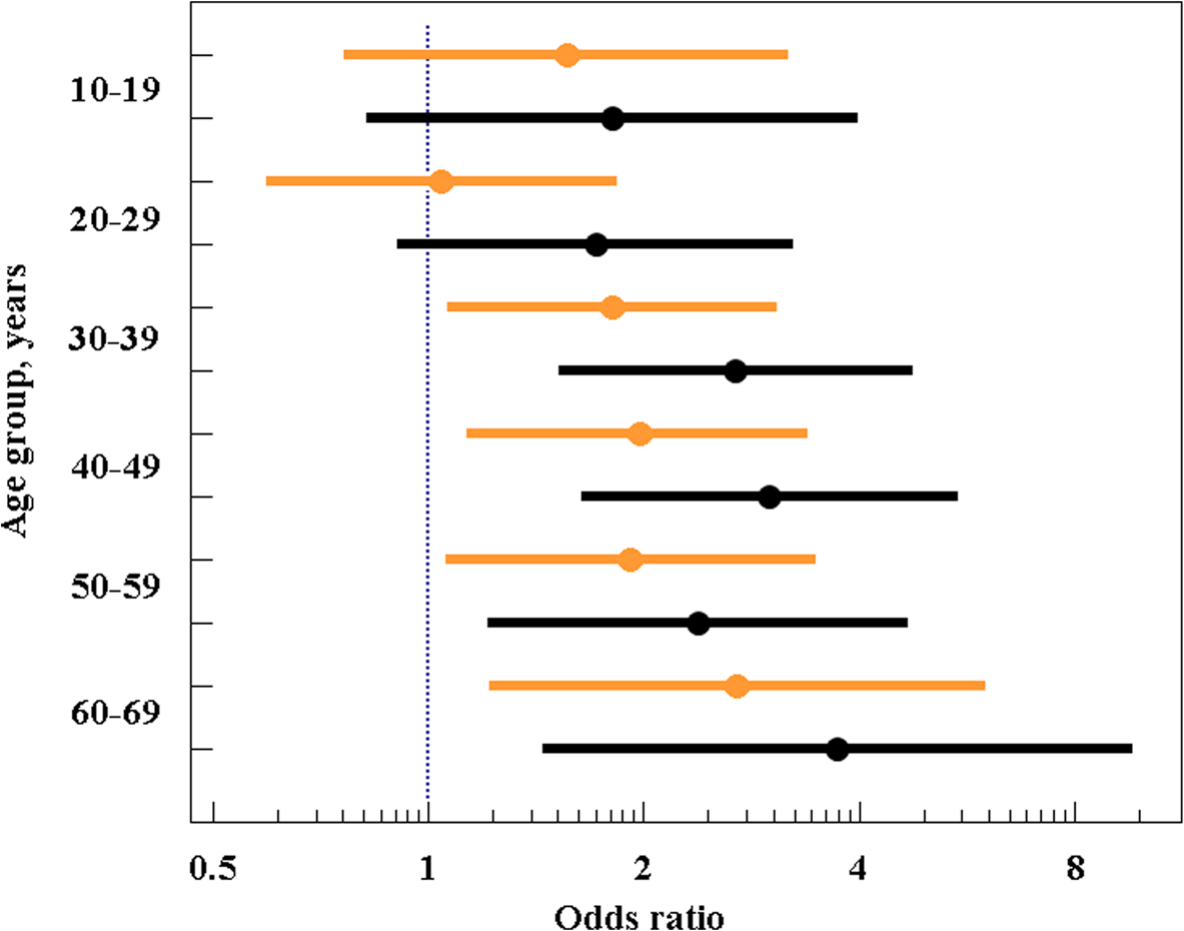
Odds ratios and 95% CIs to have F/B > 1 in males and females from different age groups compared to corresponding reference groups (ORs = 1 in both male and female 0–9-year reference groups). Female, orange; male, black

The absence of significant sex effect was further confirmed by logistic regression analysis. In the logistic regression model fitted with age and sex as covariates, no significant association was found between the age and the F/B ratio (Table 2). Accordingly, odds ratios (ORs) to have F/B > 1 tended to increase with age in both sexes, reaching maximum values of 2.7 (95% CI, 1.2–6.0) and 3.7 (95% CI, 1.4–9.6) in female and male 60–69-year age groups, respectively, compared to same-sex reference (0–9-year) age groups (Fig. 3).

**Table 2:** Logistic regression analysis of association between age and F/B ratio

Independent variables		B ± SE	Significance level, *p*	OR (95% CI)
Age	0–9	Reference		
	10–19	0.54 ± 0.27	0.042	1.72 (1.02–2.91)
	20–29	0.27 ± 0.21	0.204	1.31 (0.86–1.98)
	30–39	0.78 ± 0.19	< 0.001	2.19 (1.49–3.2)
	40–49	0.87 ± 0.2	< 0.001	2.4 (1.61–3.58)
	50–59	0.78 ± 0.22	0.001	2.19 (1.41–3.39)
	60–69	1.16 ± 0.31	< 0.001	3.19 (1.74–5.84)
Sex	Female	Reference		
	Male	−0.21 ± 0.11	0.059	0.81 (0.66–1.01)

*B* Logistic regression coefficient, *SE* Standard error

Event: F/B > 1, No event: F/B < 1

## Discussion

Microbiome is undoubtedly an important determinant of many aging-related pathological states. Among them, there are chronic inflammation [22], neurodegeneration [23], sarcopenia [24], osteoporosis [25], obesity, metabolic syndrome and type 2 diabetes [26, 27], cardiovascular disease [28] and cancer [29]. Therefore, age-associated changes in microbiota composition and function seem to be very important in the biogerontological context.

Age-related change in the F/B ratio could be particularly important. Firmicutes and Bacteroidetes are two dominant phyla representing together up to 90% of the total gut microbiota [30]. The F/B ratio has been suggested as an important index of the health of gut microbiota [31]. This ratio is known to be associated with different pathological states including the aging-associated ones [32]. For instance, the association of high F/B ratio with obesity and metabolic syndrome has been observed repeatedly [33]. Mechanistically, it may be also associated with aging-related processes determining human health status. In particular, it has been shown to be significantly associated with production of short chain fatty acids such as butyrate and propionate [34]. These microbiota-generated short chain fatty acids are known to substantially influence human healthspan. In particular, butyrate is important anti-inflammatory molecule acting on both enterocytes and circulating immune cells, thereby regulating gut barrier integrity, while propionate production is of importance for human health since it promotes satiety and prevents hepatic lipogenesis thereby lowering the cholesterol production [35, 36]. Moreover, the increased F/B ratio has been shown to be associated with an increased energy harvest from colonic fermentation [37].

However, even despite the obvious clinical importance of the gut microbiota composition, its age-related dynamics, including dynamics of F/B ratio, was evaluated only in few small-size studies, and only one available study was exclusively focused on the examination of this dynamics [38]. In this research, the F/B ratio was found to sharply increase from infancy to adulthood, and then just as sharply decreased from adulthood to old age. More specifically, F/B ratios were 0.4, 10.9 and 0.6 in infants (3 weeks to 10 months old, *n* = 21), adults (25–45 y, *n* = 21) and elderly (70–90 y, *n* = 20), respectively. In the Korean urbanized town communities, F/B ratio has been shown to be significantly higher in adults (40–69 y, *n* = 40) than in children (0–8 y, *n* = 22) [39]. The decrease in relative abundance of Firmicutes and increase in relative abundance of Bacteroidetes have been observed in the elderly (70–85 y, *n* = 18) compared to young adults (21–39 y, *n* = 14) [40]. The F/B ratio was also found to be lower in elderly subjects (65 y and older, *n* = 161) when compared to young adults (28–46 y, *n* = 9) in the study by Claesson et al. [41]. It has been speculated that such changes in microbiome composition may reflect the gradual decline of organ function and ability to maintain barrier integrity in elderly [42, 43]. In the study by Biagi et al. [44], F/B ratios were estimated as 3.6, 5.1, and 3.9 in young adults (25–40 y, *n* = 20), elderly (63–76 y, *n* = 22) and centenarians (99–104 y, *n* = 21), respectively. The differences among the age groups were, however, not statistically significant. More recently, higher proportions of Bacteroidetes and lower proportions of Firmicutes were observed in centenarians (95–108 y, *n* = 30) living in Longevity Villages of South Korea compared to those in elderly (67–79 y, *n* = 17) living in the same villages [45]. Summarizing findings from these studies and comparing them with our results, it can be concluded that our findings corroborate data presented in previous publications. In our study, consistently with previous findings, F/B ratio tended to increase with age from childhood to elderly age. The opposite trend to decrease F/B ratio has been observed in a more advanced age group which were not included in the overall analysis due to its small sample size. Such a small sample size was due to the obvious difficulty in recruiting healthy people that met the criteria for inclusion (e.g., absence of serious health problems or multidrug consumption) among the old elderly.

There are certainly multiple factors contributing to age-related phylogenetic changes in the microbiota composition. Undoubtedly, diet is among the most important factors affecting the composition of the human intestinal microbiome. Indeed, there is convincing evidence that Western-style diet characterized by high intakes of saturated fat and sugar is associated with a Bacteroides-dominant microbiome, while the more traditional high-fiber diet rich in fermented foods, vegetables and fish is associated with a high Firmicutes abundance [46]. Since the last (pro-healthy) dietary pattern is more common in older age groups [47], age-related differences in this pattern may likely explain the trend to increase in the F/B ratio with age observed in our and other studies. Other potential explanations can include influence of hormones such as sex hormones and their changing levels over age [48], impact of immunosenescence and related systemic inflammation (inflamm-aging) [49], and also repeated antibiotic interventions [50]. Moreover, the observed microbiota changes may be likely associated with age-related alterations in body mass index (BMI) found to be steadily raised in various countries across age groups until the early old age (appr. Age 60) and decreased thereafter (see, e.g., ref. [51–54]). Importantly, the association between BMI and composition of gut microbiota at different taxonomic levels, including the F/B ratio, has been well established in many populations including the Ukrainian one [55]. Therefore, age-related dynamics of BMI could be an important factor affecting the association between age and F/B ratio. Indeed, it can be suggested that age trends in F/B ratio observed in our research and in studies conducted in other populations may mirror respective age trends in BMI. We did not have data on the height and weight for participants of present study. Howerer, given that BMI might be an important confounder of the association between age and F/B ratio, we plan to include such data in our further research.

The primary strength of present study compared with prior studies on the topic is the relatively large sample size. One exception is the oldest age group (70+). The small sample size in this age group allows only preliminary conclusions. Therefore, we plan to recruit more old people for further studies. The other limitation of our research is the cross-sectional design used which did not allow us to draw definitive conclusions regarding the causal relationships between age and gut microbiota composition. One more direction for our future research is to investigate more diverse microbiota taxonomic levels. In present study, we evaluated relative abundances of the most abundant phyla, such as Firmicutes, Bacteroitedes and Actinobacteria, representing about 90% of the total bacterial population in studied sample. It is known, however, that increment in the relative abundance of Gram-negative bacteria such as Proteobacteria is one of the most important detrimental age-related changes in the human gut microbiota composition, since these Gram-negative bacteria secrete lipopolysaccharides that can cause inflammation in the gut [56]. Therefore, we plan to evaluate the relative abundance of Proteobacteria, among other Gram-negative bacteria, in our further studies.

When discussing potential mechanisms contributing to the observed age-related phylogenetic changes in the composition of microbiota, it needs to consider that it is apparently difficult to distinguish correlation from causality in the relationships observed [48]. Indeed, the microbiota and the host influence each other reciprocally, and different constituents of the microbiota interact in various ways among themselves over a human life course. Therefore, more in-depth studies are needed for a better understanding of mechanisms underlying age-related dynamics of human microbiota composition.

## Conclusions

In conclusion, data from our study indicate that composition of the human intestinal microbiota at the level of major microbial phyla can significantly differ across age groups. In both sexes, the F/B ratio tended to increase with age from 0 to 9-year to 60–69-year age groups.

## Methods

### Study population

Fecal samples were obtained from 1550 subjects residing in Ukraine and visited medical clinics in Dnipro and Kyiv for laboratory examinations and consultations throughout the time period from 19 May, 2017 to 16 March, 2020. The main demographic characteristics of the study population are provided in Table 3. Before enrollment, written informed consent has been obtained from each study participant to provide a stool sample and to an availability of stored sample for additional assays. The exclusion criteria were specified as follows: (a) refusal to give an informed consent; (b) serious health problems such as recent surgery, current presence of infectious disease or cancer, types 1 diabetes, mental illness or poorly controlled type 2 diabetes; (c) consumption of prebiotics, probiotics and antibiotics within 3 months before the enrollment date.

**Table 3:** Basic characteristics of the study population

Age group	Female, n (%)	Male, n (%)	All, n (%)
0–9	69 (45.1%)	84 (54.9%)	153
10–19	55 (59.1%)	38 (40.9%)	93
20–29	173 (67.6%)	83 (32.4%)	256
30–39	308 (68.3%)	143 (31.7%)	451
40–49	218 (67.9%)	103 (32.1%)	321
50–59	126 (66.3%)	64 (33.7%)	190
60–69	43 (64.2%)	24 (35.8%)	67
70+	11 (57.9%)	8 (42.1%)	19
**Total**	1003	547	1550

### Sample collection and DNA extraction

Fresh stool samples have been provided once by each of the study participants in a stool container on site. All fecal samples were collected and frozen immediately upon defecation. The collected samples were stored at −20 °C for about 1 week until DNA isolation. DNA was extracted from frozen aliquot (100 mg) of fecal sample using the phenol-chloroform method by protocol provided by Zhang et al. [57]. DNA samples have been stored in elution buffer (200 μl per sample). The DNA quality and quantity were evaluated by NanoDrop ND-8000 (Thermo Scientific, USA). Solutions containing DNA concentration less than 20 ng and/or an A 260/280 less than 1.8 were subjected to an ethanol precipitation to meet the quality standards.

### PCR amplification

PCR reactions were conducted using a real-time thermal cycler Rotor-Gene 6000 (QIAGEN, Germany) as described previously [55] with modifications. Conditions of PCR reaction were as follows: an initial denaturation for 5 min at 95 °C, 30 cycles of 95 °C for 15 s, annealing (15 s at 61.5 °C and 72 °C at 30 s), and then a final elongation at 72 °C for 5 min. Every PCR reaction contained 0.05 units/μl of Taq polymerase (Sigma Aldrich), 0.2 mM of each dNTP, 0.4 μM of each primer, 1× buffer, ~ 10 ng of DNA and water to 25 μl. All samples have been amplified with all primer pairs in triplicates. The Cts (both universal and specific) were the threshold cycles registered by the thermocycler. The average Ct values obtained for each pair have been transformed into percentages by the formula provided by Bacchetti De Gregoris et al. [58]:

$$ \mathrm{X}={\left(\mathrm{Eff}.\kern0.5em \mathrm{Univ}\right)}^{{\mathrm{Ct}}_{\mathrm{univ}}}/{\left(\mathrm{Eff}.\kern0.5em \mathrm{Spec}\right)}^{{\mathrm{Ct}}_{\mathrm{spec}}}\ast 100, $$

where Cts (both universal and specific) are the threshold cycles registered by the thermocycler. Eff. Univ refers to the calculated efficiency of universal primers (2 = 100% and 1 = 0%) and Eff. Spec is the efficiency of the taxon-specific primers. According to the equation, X represents the percentage of 16S taxon-specific copy number in a given sample. The detection of qPCR amplification products was based on the fluorescence of SYBR Green dye. The qPCR amplification efficiency was determined using multiple dilution series.

### Identification of the gut microbiota composition at the phylum level

Determination of microbiota composition at the level of major bacterial phyla was performed with quantitative real-time PCR (qRT-PCR) by a method described by Bacchetti De Gregoris et al. [56] using universal primers targeting bacterial 16S rRNA gene, as well as primers specific for Actinobacteria*,* Firmicutes and Bacteroidetes. All these primers were taken from the Bacchetti De Gregoris et al. [58] article. The primer sequences used are presented in Table 4.

**Table 4:** Primer nucleotide sequences used for qRT-PCR assay

Phylum	Primer nucleotide sequence	
	Forward	Reverse
Actinobacteria	Act920F3 TACGGCCGCAAGGCTA	Act1200R TCRTCCCCACCTTCCTCCG
Firmicutes	928F-firm TGAAACTYAAGGAATTGACG	1040FirmR ACCATGCACCACCTGTC
Bacteroidetes	798cfbF CRAACAGGATTAGATACCCT	cfb967R GGTAAGGTTCCTCGCGCTAT
*16S rRNA gene*	926F AAACTCAAAKGAATTGACGG	1062R CTCACRRCACGAGCTGAC

### Statistical analysis

All statistical analyses were performed with the MedCalc statistical software version 19.2 (MedCalc Software Inc., Broekstraat, Belgium, 1993–2020). The normality of the distribution of quantitative variables was assessed by Shapiro–Wilk test. The variables studied did not follow a normal distribution, so non-parametric methods were applied in analyses. Numerical data have been expressed as medians and interquartile ranges (25th to 75th percentiles) or 95% confidence intervals (CIs), as appropriate. To identify the statistical difference among age groups, median abundances of each phylum have been compared by Kruskal-Wallis test. Pairwise multiple comparisons between groups were performed by post-hoc Dunn’s test. A multivariate logistic regression analysis has been applied to estimate the effects of age and sex on F/B ratio. Odds ratios (ORs) and corresponding 95% CIs were also calculated from the logistic regression model. The significance threshold was set at *p* < 0.01.

## Data Availability

The datasets used and/or analysed during the current study are available from the corresponding author on reasonable request.

## Abbreviations

F/B ratio: Firmicutes to Bacteroidetes ratio
IQR: Interquartile range
GWAS: Genome-wide association study
OR: Odds ratio
qRT-PCR: Real-time polymerase chain reaction
